# Embryonic stem cell microenvironment suppresses the malignancy of cutaneous melanoma cells by down‐regulating PI3K/AKT pathway

**DOI:** 10.1002/cam4.2207

**Published:** 2019-06-07

**Authors:** Chenjie Wang, Xiaoran Wang, Jiahui Liu, Zheqian Huang, Chaoyang Li, Ying Liu, Xuan Sang, Liu Yang, Shoubi Wang, Yaru Su, Chengxiu Liu, Yizhi Liu, Zhichong Wang

**Affiliations:** ^1^ State Key Laboratory of Ophthalmology Zhongshan Ophthalmic Center, Sun Yat‐sen University Guangzhou 510060 China

**Keywords:** anti‐tumor effects, embryonic stem cell, melanoma, microenvironment, PI3K/AKT

## Abstract

Malignant cancer cells engage in a dynamic reciprocity with the tumor microenvironment (TME) that promotes tumor growth, development, and resistance to therapy. Early embryonic blastocyst microenvironments can reverse the tumorigenic phenotype of malignant cancer cells via ameliorating of TME. It is potential to apply embryonic stem cell (ESC) microenvironment to suppress the malignant behaviors of cancer cells. This study aimed to investigate a better method and the mechanism of ESC microenvironment supplied by ESCs on suppressing the malignancy of cutaneous melanoma cells. Cutaneous melanoma cell line A2058 were cultured and divided into four groups: (a) A2058‐only (Control); (b) A2058 and ESCs continuously co‐cultured (Group One); (c) A2058 co‐cultured with daily refreshed ESCs (Group two); (d) Group one with VO‐Ohpic, inhibitor of PTEN (VO‐Ohpic Group). The results showed that, compared to control group, A2058 cells in group one exhibited decreased cellular proliferation, migration, invasiveness and vasculogenic mimicry concomitant with an increase in cell apoptosis, accompanied by down‐regulation of PI3K/AKT pathway. Besides, the above mentioned anti‐tumor effects on A2058 cells were significantly enhanced in group two but statistically weakened after administration of VO‐Ohpic compared to group one. We demonstrate that ESC microenvironment reduces the malignancy of A2058 by down‐regulating PI3K/AKT pathway. Notably, such anti‐tumor effects can be enhanced by appropriately increasing the quality and quantity of ESCs in co‐culture system. Our results suggest that ESC microenvironment could be an effective and safe approach to treating cancer.

## 1 INTRODUCTION

The tumor microenvironment (TME) acts as an extremely important protective impact of cancer cells and is being increasingly recognized as a critical component in tumor growth, progression and therapy resistance.^1^, ^2^ Therefore, it is extremely necessary to focus on amending TME, rather than struggling to wipe out cancer cells only in antitumor treatment.

Studies have documented that early embryonic blastocyst microenvironments can reverse the tumorigenic phenotype of malignant cancer cells via ameliorating of TME.^3^, ^4^, ^5^, ^6^, ^7^ However, embryonic blastocyst microenvironments cannot be applied directly in clinical practice. Aggressive cancer cells resemble embryonic stem cells (ESCs) in their ability of unlimited proliferation, self‐renew and plasticity, furthermore, both of them are influenced deeply by continuous interactions and exchanges with their respective microenvironments.^8^, ^9^ However, key regulatory factors and signaling molecules within ESCs sustain a microenvironment that preserve the equilibrium of cell self‐renewal, differentiation and apoptosis, whereas the TME lacks the appropriate negative regulatory mechanisms and induces tumorigenic behaviors in cancer cells.^8^, ^9^, ^10^, ^11^, ^12^, ^13^ Therefore, it probably helped to substitute the ESC microenvironment for embryonic blastocyst microenvironment to modify the malignant cancer cells by amending TME. However, studies using ESC‐conditioned medium or the ESC‐conditioned extracellular 3D model yield much weaker anti‐tumor effects than those observed in early embryos.^11^, ^14^ It is likely because of the lack of direct interaction between cancer cells and ESCs. In our previous study, ESCs are injected into leukemia mice and provide leukemia mice with safe and effective ESC microenvironment, which suppresses leukemic cells and enhances the mice's survival rates.^15^ These findings indicate that ESC microenvironment supplied by ESCs can exert stronger unique and specific effects in tumor cells through interactions between ESCs and cancer cells, or ESC‐derived factors deposited into the microenvironment. Thus, the present study attempts to directly co‐culture A2058 with ESCs to explore a better way and the exact molecular mechanism of ESC microenvironment on suppressing the malignancy of tumor cells.

Unlike tumor cells, the critical components that comprise the TME and their roles in tumor progression are common between different cancers.^2^ Hence, a comprehensive understanding the mechanism of ESC microenvironment on A2058 represent a promising approach to inhibit the development of diverse tumors in clinical management.

## 2 METHODS

### 2.1 Cell culture

Malignant melanoma cell line A2058, purchased from American type culture collection (ATCC, USA), were cultured in high‐glucose DMEM (Corning, USA) with 1% penicillin–streptomycin (Gibco, USA) and 10% FBS (Corning). Mouse ESCs were generously provided by Professor P. Xiang in Sun Yat‐sen University, China. Our laboratory group successfully constructed stable transfected ESC‐GFP.^15^ ESCs were plated at a density of 200/cm^2^ in mouse ESC culture medium as described.^16^ All cells culture in the present study were maintained under 5% CO_2_ at 37°C in a humidified incubator.

### 2.2 Directly co‐culture system for ESCs‐GFP and A2058

A2058 were cultured under the following four conditions:

1. Control: A2058 were cultured alone in A2058 culture medium 72 hours.
2. Group One: Co‐cultured A2058 with ESCs‐GFP at a 1:5 ratio in A2058 culture medium 72 hours directly and continuously. There are five times as many ESCs as A2058.
3. Group Two: Directly co‐cultured A2058 with ESCs‐GFP by the proportion of 1:5 in A2058 culture medium 24 hours, thereafter A2058 together with ESCs‐GFP were dissociated with TrypLE^™^ Express Enzyme (Invitrogen, USA) and counted. The total number of A2058 in mixed cells was calculated via their respective ratios, which was detected by LSRFortessa^™^ flow cytometer (BD Bioscience, USA). Added new ESCs‐GFP, five times the number of A2058, to the mixed cell suspension. Then reseeded the above cell suspension into new cell culture flasks (Corning) in appropriate number. After 24 hours, new ESCs‐GFP was added in the same way and kept in co‐culture for another 24 hours.
4. VO‐Ohpic Group: Co‐cultured A2058 with ESCs‐GFP at a ratio of 1:5 in A2058 culture medium containing 1500 nmol/L VO‐Ohpic (MedChemExpress, USA) 72 hours directly and continuously.

A2058 in co‐culture groups were isolated by FACSAria^™^ Fusion flow cytometer (BD Bioscience, USA), respectively.

### 2.3 Labeling of cells

In order to collect ESCs for analyses of OCT4 expression from cell mixture by FACSAria^™^ Fusion flow cytometer, A2058 was labeled by Vybrant^®^ DiD cell‐labeling solution (Invitrogen) according to the manufacturer's instructions. After 2 passages, the DID‐labeled A2058 (A2058‐DID) were used for co‐culture.

### 2.4 Analyses of OCT4 expression

Normal ESCs, ESCs after co‐culture with A2058‐DID 24 hours, 48 hours, and 72 hours were washed with stain buffer twice and then centrifuged at 300*g* for 5 minutes to remove the supernatant. And BD Cytofix fixation buffer was gently added and incubated for 20 minutes at room temperature (RT). Thereafter cells were washed twice and resuspended in 1X BD Perm/Wash buffer again, and incubated for 10 minutes at RT. A part of normal ESCs was taken as negative control and added to the following components to each tube as described in Table 1 to stain cells for 30 minutes in the dark at RT. All tubes were placed on the LSRFortessa^™^ flow cytometer and data recorded, respectively. The experiment was performed three times.

**Table 1:** Components for staining ESCs of OCT4

Component	Volume to add to tube labeled					
	Negative control	Isotype control	Blank control	ESCs after co‐culture with A2058		
				24 h	48 h	72 h
Permeabilized cells (at 1 × 10^7^ cells per mL)	100 μL	100 μL	100 μL	100 μL	100 μL	100 μL
Alexa Fluor^®^ 647 OCT4	—	—	20 μL	20 μL	20 μL	20 μL
Alexa Fluor^®^ 647 Isotype control	—	20 μL	—	—	—	—

Abbreviation: ESC, embryonic stem cells.

### 2.5 Cell proliferation assay

A2058 from each group was harvested and seeded into 96‐well plates (Corning, USA) at a density of 1000 cells per well. After 24 hours, 10 µL of cell proliferation and cytotoxicity assay kit‐8 (CCK‐8, Japan) was added to each well. The plates were incubated for an additional 1 hour at 37°C in a humidified incubator. The optical density (OD) values were evaluated by Thermo Scientific Fluoroskan Ascent FL (Thermo Fisher Scientific Inc) at 450 nm. Cell proliferation curves were generated according to the OD values for 5 days. The experiment was typically evaluated three independent times in triplicate.

### 2.6 Colony formation assay

Approximately, 300 A2058 in each group were plated in triplicate into 6‐well plates, respectively. After 7 days of colony growth, the colonies were fixed with 4% formaldehyde for 20 minutes, stained with crystal violet (0.1%) for 10 minutes at RT, and counted. The assay was performed three independent times in triplicate.

### 2.7 Cell cycle analysis

A2058 in each group was harvested and adjusted to 1–5 × 10^5^/mL and fixed in 70% ice‐cold ethanol at −20°C for 2 hours. Subsequently, the cells were added RNA enzyme (Sigma–Aldrich) and incubated at 37°C for 30 minutes, followed by staining with propidium iodide (Sigma–Aldrich) for 30 minutes in the dark at RT. LSRFortessa^™^ flow cytometer was used to detect the cell cycle profiles. The experiment was replicated at least three times.

### 2.8 Cell apoptosis analysis

A2058 in each group was, respectively, stained with Annexin V‐APC/7‐AAD Apoptosis Detection Kit (KeyGEN BioTECH, China) according to the manufacturer's instruction. Apoptosis assay was evaluated by LSRFortessa^™^ flow cytometer. The experiment was replicated at least three times.

### 2.9 Wound healing assay

A2058 from each group was, respectively, inoculated into 96‐well culture plates at a density of 5 × 10^4^ cells/well until to form a monolayer with 90% confluency next day in a A2058 culture medium. A sterile plastic micropipette tip was used to create a straight‐edged, cell‐free scratch across the cell monolayer in each well, the monolayer was washed to remove cell debris and added serum‐free medium. Wound closure of the monolayered cells was monitored at the time of wounding (0 hour), and after 6 and 12 hours by taking sequential digital photographs at ×100 magnification, using inverted phase contrast microscope (Carl Zeiss Meditec AG, Jena, Germany) at the same position. The distance was measured and calculated for assessing the cellular capabilities of migration. The assay was performed three independent times.

### 2.10 Migration and invasion assays

For migration assay, about 1 × 10^5^ A2058 in each group were resuspended in 200 μL serum‐free medium and seeded into the upper chambers of the trans‐well (8.0 μm pore‐size, Corning). Then medium with 10% FBS was added to the bottom chambers. After incubation for 3 hours, the cells on the upward side of the chamber were removed using a cotton swab, and the migrated cells in another chamber were fixed in paraformaldehyde (4%) and stained with crystal violet (0.1%) for 15 minutes at RT. The stained cells were photographed using an inverted phase contrast microscope at ×100 magnification and quantified at an average of three fields per membrane under microscopic inspection.

Similar to the migration assay, about 1 × 10^5^ A2058 in each group were seeded into the upper chamber of the matrigel‐coated chambers (BD). The remaining steps are consistent with the migration assay.

All experiments were performed three independent times in triplicate.

### 2.11 Matrigel tube formation assay

Here, 96‐well tissue culture dishes were coated with 30 μL of Matrigel (BD Biosciences) and were incubated at 37°C for at least 30 minutes before use, allowing Matrigel to polymerize. A2058 was seeded onto the plates at 1.6 × 10^5^ cells per well in triplicate and vasculogenic mimicry (VM) networks were imaged at ×100 magnification by inverted phase contrast microscope at 12 hours. The assay was performed three independent times.

### 2.12 Reverse transcription‐quantitative polymerase chain reaction analysis

The total RNA of A2058 in each group was extracted based on the manufacturer's protocol of RNeasy Plus Mini Kit (QIAGEN, Valencia, CA, USA). RNA concentration was detected by NanoDrop 1000TM (Thermo Fisher Scientific, Waltham, MA, USA). An equivalent amount of RNA was reverse transcribed to cDNA using the SYBR PrimeScript^™^ RT Master Mix (Takara Bio, Otsu, Japan). Referring to the instructions, real‐time PCR was processed on LightCycler 480 (Hoffman‐La Roche Ltd., Basel, Switzerland), using SYBR Premix Ex Taq Kit (Takara Bio, Otsu, Shiga, Japan). Each reaction system was 20 µL, including SYBR@Premix Ex TaqII (10 µL), ROX Reference Dye (0.4 µL), Forward Primer (0.4 µL), Reverse Primer (0.4 µL), cDNA (2 µL), and DEPC‐treated water (6.8 µL) (Invitrogen^™^). GAPDH was chosen as the internal reference. All reactions were run in triplicate. The primer sequences were listed in Table 2.

**Table 2:** Primer sequences for RT‐qPCR analysis

Genes	Primer sequences
β‐actin	Forward: 5′‐TCATCACCATTGGCAATGAG‐3′
	Reverse: 5′‐CACTGTGTTGGCGTACAGGT‐3′
CD44	Forward: 5′‐ATGGGTTCATAGAAGGGCACG‐3
	Reverse: 5′‐TGTCATACTGGGAGGTGTTGGA‐3
PTEN	Forward: 5′‐CTCAGCCGTTACCTGTGTGTG‐3
	Reverse: 5′‐AGGTTTCCTCTGGTCCTGGTA‐3
AKT1	Forward: 5′‐ATTGTGAAGGAGGGTTGGCTG‐3
	Reverse: 5′‐CCGCTCCTTGTAGCCAATGAA‐3
AKT3	Forward: 5′‐GGAGAGGAAGAGATGGATGCCT‐3
	Reverse: 5′‐CCACTTGCCTTCTCTCGAACC‐3
mTOR	Forward: 5′‐CCCTCCATCCACCTCATCAGT‐3
	Reverse: 5′‐CGCCAAGACACAGTAGCGAAT‐3
Cyclin B1	Forward: 5′‐GGTTGTTGCAGGAGACCATGT‐3
	Reverse: 5′‐AACATGGCAGTGACACCAACC‐3
Cyclin D1	Forward: 5′‐TTCATTTCCAATCCGCCCTCC‐3
	Reverse: 5′‐TGTGAGGCGGTAGTAGGACAG‐3
MMP1	Forward: 5′‐CTCTGGAGTAATGTCACACCTCT‐3
	Reverse: 5′‐TGTTGGTCCACCTTTCATCTTC‐3
MMP9	Forward: 5′‐GGGACGCAGACATCGTCATC‐3
	Reverse: 5′‐TCGTCATCGTCGAAATGGGC‐3
Ve‐cad	Forward: 5′‐TGTTCACGCATCGGTTGTTCA‐3
	Reverse: 5′‐TACATGACAGAGGCGTGGTCT‐3

Abbreviations: RT‐qPCR, Reverse transcription‐quantitative polymerase chain reaction; Ve‐cad, vascular endothelial‐cadherin.

### 2.13 Immunofluorescence staining

The cells were fixed with paraformaldehyde (4%) for 20 minutes, permeabilized with 0.1% Triton X‐100 in PBS for another 15 minutes, and blocked with goat serum (BOSTER Biological Technology co. ltd). Cells were stained with a primary antibody as described in Table 3 overnight at 4 °C, washed 3X in PBS, and then incubated with Alexa Fluor Plus 647 donkey anti‐rabbit IgG secondary antibody (Invitrogen^™^, # A32795) at a dilution of 1:1000 for 2 hours at RT. The nucleus was counterstained with Hoechst 33258 (Invitrogen^™^, # H3569) at a dilution of 1:1000 for 15 minutes. Images were taken with a laser scanning confocal microscope (LSM 510 META; Carl Zeiss, Hamburg, Germany) at 50× magnification.

**Table 3:** Main antibodies used in Immunofluorescence (IF) staining and Western blotting (WB)

Antibody	Source	Dilution (application)
anti‐PTEN	abcam #ab32199	1:100 (WB)
		1:10000 (IF)
anti‐PI3K	abcam #ab186612	1:2000 (WB)
		1:1000 (IF)
anti‐AKT1/2/3	abcam #ab179463	1:10000 (WB)
		1:100 (IF)
anti‐mTOR	abcam #ab2732	1:1000 (IF)
anti‐Cyclin B1	abcam #ab181593	1:500 (WB)
		1:2000 (IF)
anti‐Cyclin D1	abcam #ab134175	1:50 (WB)
		1:10000 (IF)
anti‐MMP1	abcam #ab52631	1:100 (WB)
		1:5000 (IF)
anti‐MMP9	abcam #ab76003	1:250 (WB)
		1:1000 (IF)
anti‐Ve‐cad	abcam #ab33168	1:200 (WB)
		1:1000 (IF)

Abbreviation: Ve‐cad, vascular endothelial‐cadherin.

### 2.14 Western blotting

Protein was extracted and its concentration was detected using the bicinchoninic acid protein assay (Thermo Fisher Scientific, Inc). Equal quantities of protein samples were loaded onto sodium dodecyl sulfate‐polyacrylamide gel electrophoresis for electrophoresis and separated. The gel proteins were blotted onto a polyvinylidene difluoride membrane (Millipore, Billerica, MA, USA). The transferred membranes were blocked in 7% nonfatty milk for 2 hours at RT, incubated at 4°C overnight with primary antibodies as described in Table 3. After being washed thrice with TBS, the membranes were treated with diluted secondary antibody (1:2,000, cat. no. ab6789, Abcam) for 2 hours at RT. The localization of antibodies was detected using the ECL Western blotting kit (Beyotime, Beijing, China) according to the manufacturer's protocols. Here, β‐actin was employed as the internal reference.

### 2.15 Statistical analysis

All data were presented as mean ± standard deviation (SD) of three or more independent experiments. Differences between groups were analyzed using Kruskal‐Wallis 1‐way ANOVA conducted with SPSS 23.0 statistical software (IBM, USA) and *P*‐values < 0.05 were considered statistically significant.

## 3 RESULTS

### 3.1 ESCs gradually differentiated in the co‐culture system with cutaneous melanoma cells over time

**Figure 1:**
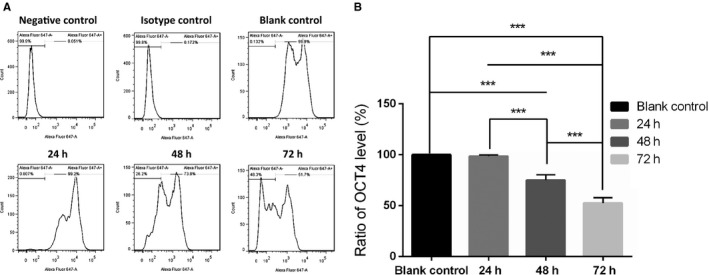
OCT4 expression of ESCs after co‐culture with A2058 gradually decreased along with the time. A, Representative images of OCT4 expression of ESCs in the negative control, isotype control, blank control, after co‐culture with A2058 24 h, 48 h, 72 h detected by flow cytometry, respectively. B, The comparison of OCT4 expression of ESCs in blank control, after co‐culture with A2058 24 h, 48 h, 72 h. Data presented as the mean with the SD. ***P < 0.0010. ESCs, embryonic stem cells

ESCs have been defined as self‐renewing, pluripotent cells, and expressed high levels of Oct4 over the extended culture.^17^ Oct4 appears to be imperative to retain stemness in mouse ESCs, as evidenced by its down‐regulation upon cellular differentiation.^18^ To understand whether the phenotype of ESCs would change in co‐culture with A2058 over time, we detected the expression of OCT4 in ESCs using flow cytometry. As shown in Figure 1, the ratio of OCT4 expression in normal ESCs, ESCs after co‐culture with A2058 for 24 hours, 48 hours, 72 hours were 100%, 98.5 ± 1.48%, 75.0 ± 5.30%, and 52.5 ± 5.44%, respectively. The OCT4 expression in ESCs after co‐culture with A2058 24 hours was not statistically decreased compared to normal ESCs, however, it significantly declined in ESCs after co‐culture 48 hours or 72 hours. These findings indicated that ESCs gradually differentiated after co‐culture with A2058 along with time in A2058 culture medium.

### 3.2 ESC microenvironment induced the anti‐proliferative and pro‐apoptotic effects on cutaneous melanoma cells

**Figure 2:**
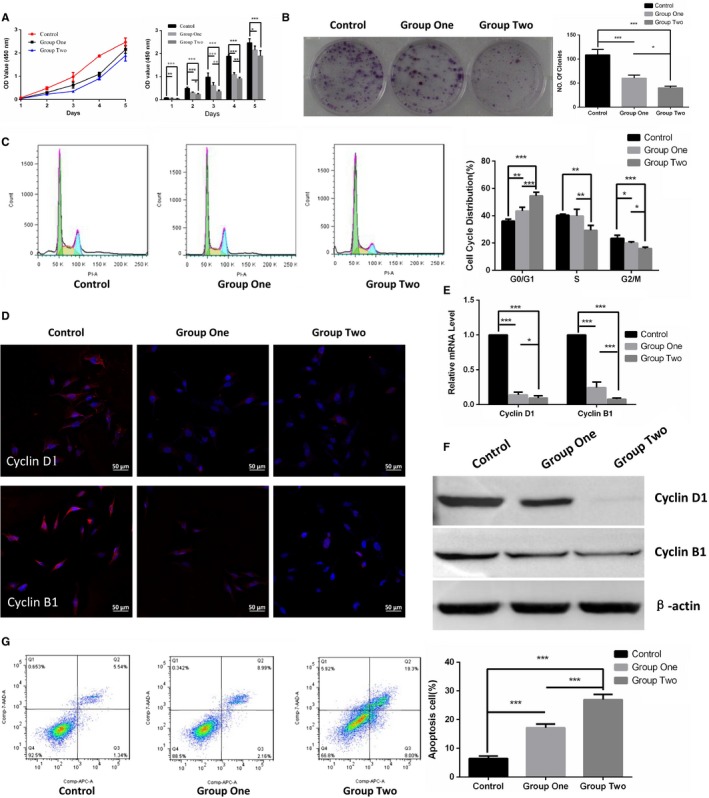
ESC microenvironment inhibited cell growth coupled with down‐regulation of Cyclin D1 and Cyclin B1 and promoted cell apoptosis in A2058. A, Cell growth kinetic curve (left panel) and quantified graph (right panel) of cell proliferation assay were performed to validate the cell proliferation of A2058 in control, group one and group two. B, The colony formation assays of A2058 were displayed in representative images (left panel) and quantified in graph (right panel) to detect the cell growth potential of A2058. C, Representative images of the cell cycle analysis (left panel) revealed the cell cycle distribution of A2058, and the graph (right panel) showed quantification for each cell cycle phase. D, Representative immunofluorescence staining of Cyclin D1 (upper panel) and Cyclin B1 (lower panel) was detected by laser scanning confocal microscope. Red represents Cyclin D1 or Cyclin B1, and blue represents nuclear DNA staining by Hoechst 33258. E, The comparison of mRNA levels of cell cycle‐relative Cyclin D1 and Cyclin B1 were performed using RT‐qPCR. Data are normalized to GAPDH. F, The expression of Cyclin D1 and Cyclin B1 were examined by WB. β‐actin was used as a loading control. G, Representative images of the cell apoptosis analysis using flow cytometry (left panel) and the graph (right panel) illustrated the quantification of apoptotic A2058. Data presented as mean ± SD. *P < 0.05, **P < 0.01 ***P < 0.001. ESC, embryonic stem cell; RT‐qPCR, Reverse transcription‐quantitative polymerase chain reaction

To analyze the effect of the ESC microenvironment on cancer cell growth, we performed cell proliferation assay and colony formation assay on A2058 in control, group one and group two, respectively. As shown in Figure 2A, cell proliferation assay revealed that the proliferation of A2058 was significantly decreased in group one and group two compared with that in control. Meanwhile, the proliferation of A2058 was suppressed more in group two than in group one. Additionally, results in colony formation assays followed a similar trend as CCK‐8 assays in Figure 2B.

We further explored the mechanism underlying the regulation of cell growth by detecting the cell cycle distribution and apoptosis of A2058. As illustrated in Figure 2C, A2058 accumulated more in the G0/G1 phase in group one and group two than in control. Analogously, more A2058 were distributed in G0/G1 phase in group two than group one. Furthermore, these observations were consistent with the down‐expression of cell cycle‐related Cyclin D1 and CyclinB1, detected by immunofluorescence staining in Figure 2D, reverse transcription‐quantitative polymerase chain reaction (RT‐qPCR) in Figure 2E and WB in Figure 2F. In parallel, the apoptotic cells in group one and group two significantly increased, as compared with that in control. Additionally, the apoptotic cells increased more observably in group two than in group one as shown in Figure 2G.

Taken together, these results demonstrated that ESC microenvironment supplied by ESCs notably inhibited cell proliferation and promoted cell apoptosis on cutaneous melanoma cells. What is more, such anti‐proliferative and pro‐apoptotic effects could be enhanced significantly by daily refreshing ESCs in the co‐culture system.

### 3.3 ESC microenvironment reduced cellular capabilities of migration and invasion of cutaneous melanoma cells

**Figure 3:**
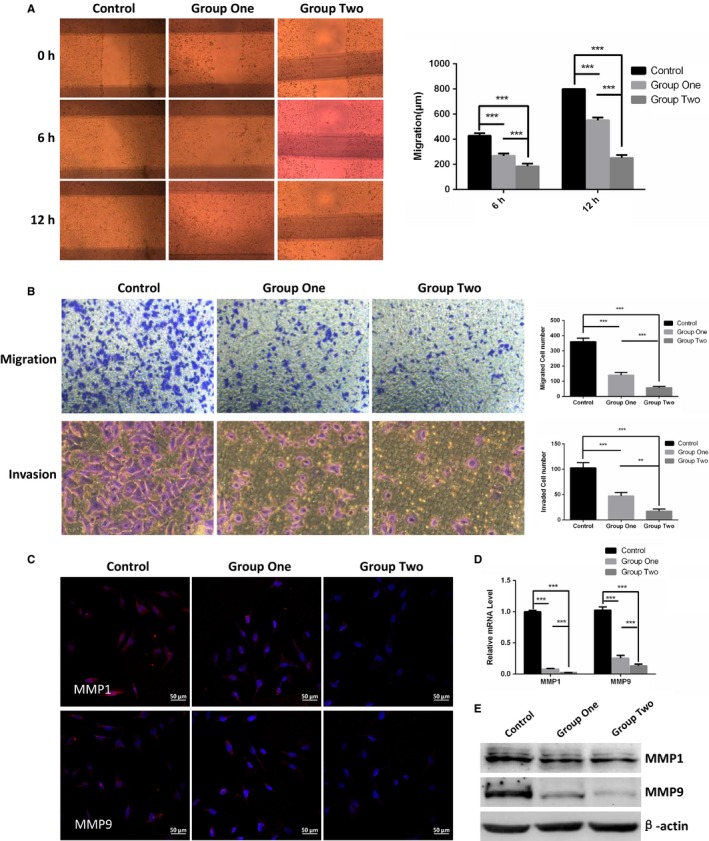
ESC microenvironment impeded invasive and migratory abilities concomitant with down‐regulation of MMP1 and MMP9 in A2058. A, Representative images of wound‐healing assay (left panel) and the migration distances (right panel) were measured to study the cellular capabilities of motility in control, group one and group two. B, Representative micrographs (left panel) and quantified graph (right panel) of migration and invasion assays were performed to detect the cell migratory and invasive abilities of A2058. C, Representative immunofluorescence staining of MMP1 and MMP9 was detected by laser scanning confocal microscope. D, The comparison of mRNA levels of MMP1 and MMP9 were performed using RT‐qPCR. Data are normalized to GAPDH. E, The expression of MMP1 and MMP9 were examined by WB. β‐actin was used as a loading control. Data presented as mean ± SD. **P < 0.01 ***P < 0.001. ESC, embryonic stem cell; RT‐qPCR, Reverse transcription‐quantitative polymerase chain reaction

To investigate the impact of ESC microenvironment in cutaneous melanoma cells migration and invasion, wound healing assay, migration and invasion assays were conducted in control, group one and group two. As illustrated in Figure 3A, the migration distance in group one and group two are all significantly shorter than in control after wounding 6 hours and 12 hours. Apparently, the migration distance was reduced more notably in group two than in group one. After wounding 12 hours, A2058 in control had completely crossed scratch zone, while the group one and group two still had the cell‐free zone. Results in migration and invasion assays in Figure 3B exhibited that the migratory and invasive properties of A2058 were also obviously suppressed compared with cells in control. Besides, migratory and invasive properties of cells in group two was decreased more than group one.

We further analyzed MMP1 and MMP9 which are important members of the matrix metalloproteinases and target the degradation and proteolytic process of extracellular matrix components. As displayed in Figure 3C‐E, the mRNA and protein levels of MMP1 and MMP9 in A2058 were all significantly decreased after co‐culture. Meanwhile, the MMP1 and MMP9 expression of A2058 in group two were decreased more than that of in group one.

Collectively, our data supported the notion that ESC microenvironment supplied by ESCs suppressed migration and invasion abilities in cutaneous melanoma cells. In addition, the migratory and invasive potential of melanoma cells could be further weakened by appropriately increasing the quality and quantity of ESC in the co‐culture system.

### 3.4 ESC microenvironment inhibited VM in cutaneous melanoma cells

**Figure 4:**
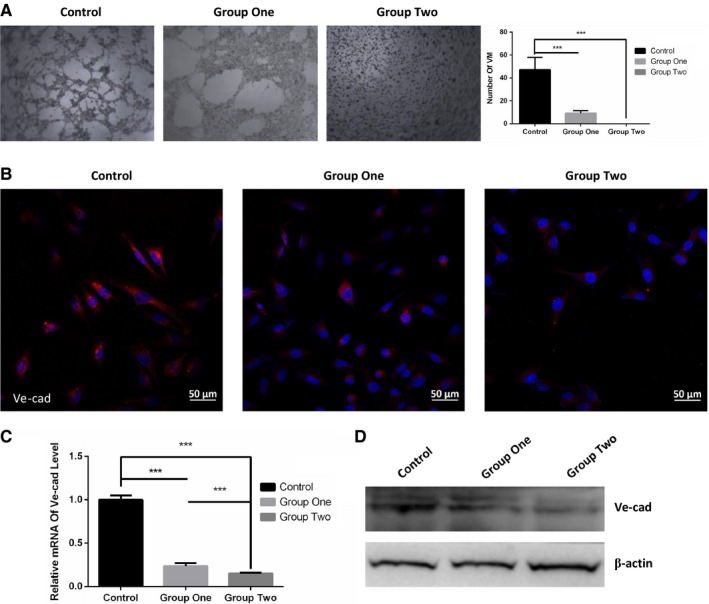
ESC microenvironment blocked VM formation, coupled with down‐regulation of Ve‐cad. A, Representative images (left panel) and quantification (right panel) of VM in A2058 using phase‐contrast microscopy. B, Representative immunofluorescence staining of Ve‐cad was detected by laser scanning confocal microscope. C, The comparison of mRNA levels of Ve‐cad using RT‐qPCR in control, group one and group two. Data are normalized to GAPDH. D, The expression of Ve‐cad were examined by WB. β‐actin was used as a loading control. Data presented as mean ± SD. ***P < 0.001. ESC, embryonic stem cell; RT‐qPCR, Reverse transcription‐quantitative polymerase chain reaction; Ve‐cad, vascular endothelial‐cadherin

Vasculogenic mimicry is uniquely patterned vasculogenic‐like networks mediated by highly aggressive tumor cells and predict stronger invasiveness in cutaneous melanoma cells.^19^, ^20^ As displayed in Figure 4A, after co‐culture with ESCs, A2058 formed less tubular structures than control, more remarkable, A2058 in group two failed to form patterned networks. Vascular endothelial‐cadherin (Ve‐cad) has a strong contributory role in melanoma VM.^21^ Consistent with VM, both mRNA and protein levels of Ve‐cad of A2058 were significantly restrained after co‐culture compared to the control as shown in Figure 4B‐D. Similarly, the Ve‐cad expression was reduced more in group two than that in group one.

In conclusion, ESC microenvironment remarkably inhibited or even disrupted VM, accompanied by down‐regulating Ve‐cad in cutaneous melanoma cells.

### 3.5 ESC microenvironment reduced the malignancy of cutaneous melanoma cells by down‐regulating PI3K/AKT signaling pathway

**Figure 5:**
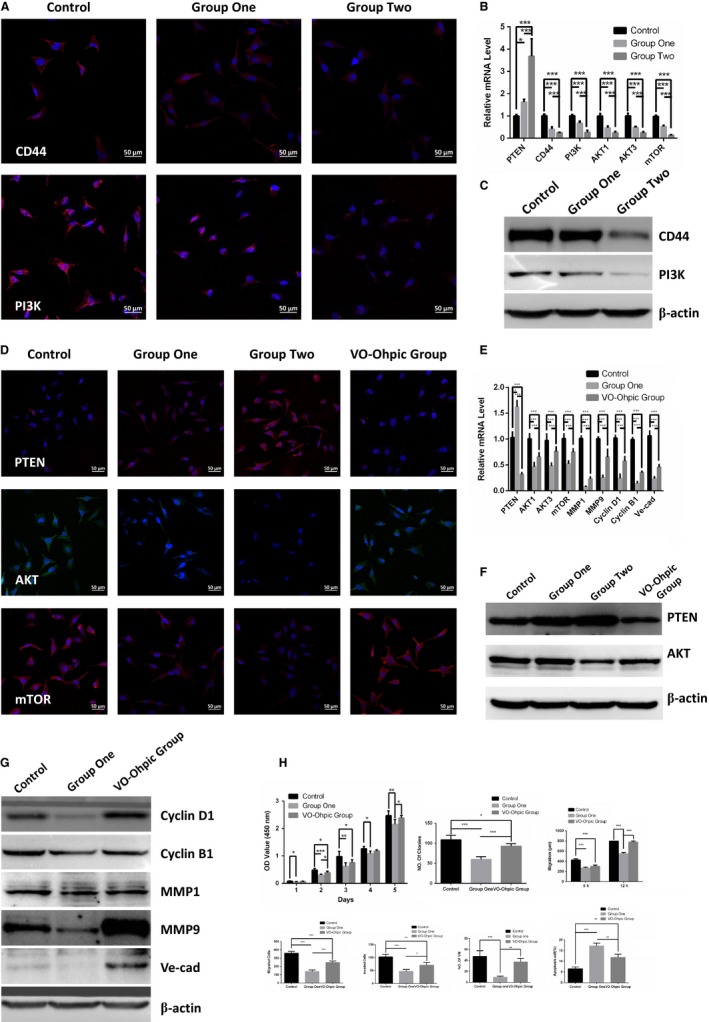
ESC microenvironment reduced the malignancy of A2058 by down‐regulating PI3K/AKT pathway. A, Representative immunofluorescence staining of CD44 and PI3K of A2058 in control, group one and group two were detected by laser scanning confocal microscope. B, The comparison of mRNA expression of PTEN, CD44, PI3K, AKT1, AKT3 and mTOR on in control, group one and group two. Data are normalized to GAPDH. C, WB analysis expressing CD44 and AKT in control, group one and group two. β‐actin was used as a loading control. D, Representative immunofluorescence staining of PTEN, AKT and mTOR in control, group one, group two and VO‐Ohpic Group. E, The comparison of mRNA expression of PTEN, AKT1, AKT3, mTOR, MMP1, MMP9, Cyclin D1, Cyclin B1, Ve‐cad in control, group one and VO‐Ohpic Group. F, The expression of PTEN and AKT were examined by WB in control, group one, group two and VO‐Ohpic Group. G, WB analysis expressing Cyclin D1, Cyclin B1, MMP1, MMP9 and Ve‐cad on A2058 in control, group one and VO‐Ohpic Group. Data presented as mean ± SD. H, Quantification and comparison of cell proliferation, migration, invasion and VM tube formation on A2058 in control, group one and VO‐Ohpic Group. *P < 0.05, **P < 0.01 ***P < 0.001. ESC, embryonic stem cell. Ve‐cad, vascular endothelial‐cadherin

The P13K/AKT signaling pathway is the classical signaling pathway that regulate human tumor development.^22^ To better characterize whether ESC microenvironment regulate the malignancy of cutaneous melanoma cells by PI3K/AKT pathway, we examined the expression of key components of the PI3K/AKT pathway in A2058. The mRNA and protein levels of CD44, PI3K, AKT1, AKT3, and mTOR in A2058 in Figure 5A‐F were notably down‐regulated after co‐culture with ESCs. Even more, the PI3K/AKT pathways are much more suppressed in group two than in group one. To further substantiate the specificity of the P13K/AKT pathway on the malignancy of cutaneous melanoma cells, the results showed that the administration of the VO‐Ohpic, inhibitor of PTEN, statistically decreased PTEN and enhanced the expression of AKT1, AKT3, mTOR, Cyclin D1, Cyclin B1, MMP1, MMP9, and Ve‐cad of A2058 in Figure 5D‐G. Analogously, the VO‐Ohpic obviously strengthened cell proliferation, migration, invasion, VM, and inhibited cell apoptosis in Figure 5H.

These results suggested that the ESC microenvironment significantly inhibited the malignant behavior of A2058 through down‐regulating of the PI3K/AKT signaling pathway and such anti‐tumor effects could be enhanced by appropriately increasing the quality and quantity of ESCs in the co‐culture system. What is more, the above‐mentioned anti‐tumor effects could be remarkably weakened by activating the PI3K/AKT pathway.

## 4 DISCUSSION

Malignant melanoma is one of the fastest growing cancers worldwide and may occur in any part of the body, most common in the skin, and is deemed to be the deadliest form of skin cancer.^23^, ^24^, ^25^, ^26^, ^27^ Tumors in distant organs are often unresectable, besides, conventional chemotherapy is low efficiency and have serious side effects. Although immunotherapies and targeted therapies represent a milestone in the treatment of metastatic melanoma, they show immune system‐related side effects, drug resistance, or even relapse.^28^

Previous studies demonstrate that the early embryonic microenvironment can reprogram malignant cancer cells toward a non‐tumorigenic phenotype without damaging normal cells.^3^, ^4^, ^5^, ^6^, ^7^ In this study, we successfully established the embryo‐like microenvironment using ESCs and demonstrated that the ESC microenvironment is capable of suppressing the tumorigenic phenotype of malignant cancer cells.

Our experiments showed that ESC microenvironment had the ability to inhibit proliferation and enhance apoptosis of cutaneous melanoma cells, coinciding with down‐regulation of Cyclin D1 and Cyclin B1, which resulted in a reduction in the percent of cells in G0/G1. It was suggested that the ESC microenvironment induces G1/S cell cycle arrest on cutaneous melanoma cells. Consistent with our findings, a series of previous studies reveal that down‐regulation of Cyclin D1 or Cyclin B1 can be induced significantly less colony‐forming, stronger anti‐proliferative effect, and pro‐apoptosis ability in different tumor cell lines.^28^, ^29^, ^30^, ^31^

Metastasis is the most essential biological characteristic of malignant cells and remains the leading cause of cancer mortality. MMP1 and MMP9 are positively related to the migration and invasion abilities on cancer cells.^32^, ^33^, ^34^, ^35^ Our data suggested that ESC microenvironment reduced the migration and invasion abilities of skin melanoma cells through down‐regulation of MMP1 and MMP9. It is generally assumed that tumors require an abundant blood supply for tumor progression.^36^, ^37^, ^38^, ^39^ The anti‐tumor approach of restraining endothelial cells from neovascularization seemed strategically sound, however, the results have been disappointing for the development of resistance.^40^ VM can provide a functional perfusion pathway for rapidly growing tumors and are regarded as one of the major causes of the development of resistance to anti‐angiogenic therapy in solid tumors.^19^, ^20^, ^41^, ^42^ VM also predict higher Tumor, Lymph Node, and Metastasis Stage (TNM Stage), stronger invasiveness, poorer prognosis, and survival outcomes.^19^ Ve‐cad is critical in melanoma VM and down‐regulation of Ve‐cad expression in the aggressive melanoma cells can abrogate their ability to form vasculogenic networks.^21^ Our results evidenced that ESC microenvironment reduced or even abrogated VM together with down‐regulation of Ve‐cad on cutaneous melanoma cells.

Functionally, the above anti‐tumor effects of ESC microenvironment were supplied by ESCs, which should maintain an undifferentiated state.^15^ However, we found ESCs gradually differentiated during the continuous co‐culture of A2058. We postulated that the differentiation of ESCs might cause negative influence on anti‐tumor effects of ESC microenvironment. Thus, we added new ESCs daily for increasing the number of undifferentiated ESCs in co‐culture system. As expected, our findings revealed, compared to continuous co‐culture, A2058 co‐cultured with added ESCs exhibited decreased cellular proliferation, migration, invasiveness, and VM concomitant with an increase in cell apoptosis. It demonstrated that the differentiation of ESCs in the co‐culture system will reduce the anti‐tumor effects of ESC microenvironment. Therefore, appropriate increase in the quality and quantity of ESCs in the direct co‐culture system can enhance anti‐tumor effects of ESC microenvironment on cutaneous melanoma cells. Although this study did not fully normalize cutaneous melanoma cells, we speculate that enhancing the quality and quantity of ESCs appropriately in the co‐culture system and prolonging the co‐culture time may obtain better anti‐tumor effects or even reverse malignant cancer cells toward normal cells.

The PI3K/AKT signaling pathway controls cell survival, proliferation, metastasis, angiogenesis, and regulates CyclinD1, CyclinB1, MMP1, MMP9, and Ve‐cad in many types of cancer.^33^, ^43^, ^44^, ^45^, ^46^, ^47^ Aberrant activation of this pathway is instrumental in tumorigenesis and tumor progression.^48^ Our findings demonstrated for the first time that ESC microenvironment suppressed the malignancy of cutaneous melanoma cells due, at least in part, to down‐regulating PI3K/AKT signaling pathway. It has been commonly agreed that the PI3K pathway is also closely related to normal physiological activities of somatic cells.^49^ Unlike drugs targeting the PI3K/ATK pathway in the clinic for cancer therapy, which have toxic side effects on normal somatic cells, the ESC microenvironment have a strong positive influence on normal cells.^50^, ^51^, ^52^, ^53^ Thus, a comprehensive understanding of the tumor‐suppressive effects of ESC microenvironment could become a powerful tool in future antitumor therapy.

## 5 CONCLUSIONS

In this study, we firstly demonstrate that ESC microenvironment supplied by ESCs inhibits malignant behaviors of cutaneous melanoma cells on cell proliferation, migration, invasion, and VM by down‐regulation of the PI3K/AKT signaling pathway. Particularly noteworthy is that appropriate increase in the quality and quantity of ESCs in the direct co‐culture system can enhance such anti‐tumor effects of the ESC microenvironment. It may shed new light on their therapeutic implications to inhibit tumor progression.

## Data Availability

The data that support the findings of this study are available from the corresponding author upon reasonable request.
